# Synthesis, Properties, and Biodegradability of Thermoplastic Elastomers Made from 2-Methyl-1,3-propanediol, Glutaric Acid and Lactide

**DOI:** 10.3390/life11010043

**Published:** 2021-01-12

**Authors:** Lamya Zahir, Takumitsu Kida, Ryo Tanaka, Yuushou Nakayama, Takeshi Shiono, Norioki Kawasaki, Naoko Yamano, Atsuyoshi Nakayama

**Affiliations:** 1 Department of Applied Chemistry, Graduate School of Advanced Science and Engineering, Hiroshima University, 1-4-1 Kagamiyama, Higashi-Hiroshima Hiroshima 739-8527, Japan; d170169@hiroshima-u.ac.jp (L.Z.); taku-kida@hiroshima-u.ac.jp (T.K.); rytanaka@hiroshima-u.ac.jp (R.T.); tshiono@hiroshima-u.ac.jp (T.S.); 2 Biomedical Research Institute, National Institute of Advanced Industrial Science and Technology (AIST), 1-8-31 Midorigaoka, Ikeda, Osaka 563-8577, Japan; n-kawasaki@aist.go.jp (N.K.); yamano.naoko@aist.go.jp (N.Y.); a.nakayama@aist.go.jp (A.N.)

**Keywords:** poly(L-lactide), triblock copolymers, thermoplastic elastomer, poly(2-methyl-1,3-propylene glutarate), biodegradability

## Abstract

An innovative type of biodegradable thermoplastic elastomers with improved mechanical properties from very common and potentially renewable sources, poly(L-lactide)-*b*-poly(2-methyl-1,3-propylene glutarate)-*b*-poly(L-lactide) (PLA-*b*-PMPG-*b*-PLA)s, has been developed for the first time. PLA-*b*-PMPG-*b*-PLAs were synthesized by polycondensation of 2-methyl-1,3-propanediol and glutaric acid and successive ring-opening polymerization of L-lactide, where PMPG is an amorphous central block with low glass transition temperature and PLA is hard semicrystalline terminal blocks. The copolymers showed glass transition temperature at lower than −40 °C and melting temperature at 130–152 °C. The tensile tests of these copolymers were also performed to evaluate their mechanical properties. The degradation of the copolymers and PMPG by enzymes proteinase K and lipase PS were investigated. Microbial biodegradation in seawater was also performed at 27 °C. The triblock copolymers and PMPG homopolymer were found to show 9–15% biodegradation within 28 days, representing their relatively high biodegradability in seawater. The macromolecular structure of the triblock copolymers of PLA and PMPG can be controlled to tune their mechanical and biodegradation properties, demonstrating their potential use in various applications.

## 1. Introduction

Nowadays, biodegradable polymers have been increasingly attracting attention to substitute non-biodegradable products as sustainable alternatives to protect the environment from plastic pollution [1]. Aliphatic polyesters, such as poly(L-lactic acid) (PLA), poly(ε-caprolactone) (PCL), poly(butylene succinate) (PBSu), poly(3-hydroxybutyrate) (PHB), and so forth, form a very important class of biodegradable polymers due to their favorable features of biodegradability and biocompatibility. Among them, PLA is one of the most promising biodegradable polymers due to its biocompatibility and availability from renewable biomass, although its hard and brittle nature limits its application.

On the other hand, ABA type triblock copolymers consisting of two immiscible blocks, a soft central block (B) and two hard terminal blocks (A), behave as thermoplastic elastomers (TPEs), which can be varied between hard plastics and soft rubbers depending on the compositions and microarchitectures [2,3]. TPEs are also known for their excellent processability and recyclability. PLA can be applied as hard terminal blocks of TPE by the introduction of a soft central block [4].

Some biodegradable and/or biobased central blocks, such as poly(1,3-propylene carbonate) [5], poly(1,5-dioxepan-2-one) [6], PCL [7], poly(ε-decalactone) (PεDL) [8] and PHB [9], were synthesized by ring-opening polymerization (ROP) and their properties were discussed by different research groups. Olsen and his coworkers reported poly(ricinoleic acid) with low glass transition temperature (*T*_g_ = −50 °C) as a soft central block in TPE [10]. This TPE showed 250 times greater elongation at break than that of pure PLA. Poly(vinylidene fluoride) was adopted as a soft central block of PLA, containing ABA type triblock copolymer, by a research team of Loos [11]. Huang and coworkers synthesized Poly(ε-caprolactone-*co*-δ-valerolactone) (PCVL) as a soft central block and reported that the mechanical property and thermal stability of PLA-*b*-PCVL-*b*-PLA TPEs were increased with the crystallization and stereocomplexation of PLA terminal blocks [12]. However, the relatively high level of stress relaxation could be considered as a major limitation in the case of amorphous copolymers. The semicrystalline aliphatic polyesters with a fairly low *T*_g_ could perform well as soft central blocks of TPE to achieve good mechanical properties [13].

To achieve fully biodegradable and biocompatible TPE, researchers have used biobased aliphatic polyesters as central blocks in PLA conjugated triblock copolymers. Various saturated aliphatic polyesters like poly(butylene adipate) [14]; random copolyesters like poly(butylene succinate-*co*-azelate) [15], poly(propylene-*co*-neopentylene succinate) [16], poly(hexamethylene (2,3-*O*-isopropylidene tartarate)) [17], and poly(hexamethylene-*co*-decamethylene adipate) [18]; and unsaturated aliphatic polyesters such as 2-butene-1,4-diol oligomers [19] have been reported in many works. Poly(but-2-ene-1,4-diyl malonate) (PBM), containing triblock copolymer with PLA hard blocks, has been reported to be of potential for biomedical application with customized degradation profile [20]. One of our previous studies reported poly(ε-caprolactone-*co*-DL-lactide) as a potential soft central block in PLA conjugated ABA type triblock copolymer to improve the mechanical property with notably high elongation at break of ≈2800% [4]. One research team has synthesized poly(propylene glutarate) as a soft central block of ABA type triblock copolyester composed of PLA and reported typical elastomeric behavior and improved toughness of PLA [21].

If 2-methyl-1,3 propanediol (MP) could be considered as monomers for synthesizing aliphatic polyesters as soft blocks, the methyl groups introduced from MP could help to disturb the crystallization of polymers [22,23,24,25,26,27]. The incorporation of methyl-branched units into PBS lowered its crystallinity to a considerable extent [28].

In our recent work, poly(2-methyl-1,3-propylene succinate) (PMPS) from succinic acid and MP was synthesized and adopted as a soft central block of the PLA-based triblock copolymers, PLA-*b*-PMPS-*b*-PLA. This TPE showed significantly lower tensile modulus and higher elongation at break than those of homopoly(L-lactide). Enzymatic and seawater degradability test were also performed, keeping in mind the increasing demand of biodegradable polymers to save the world and marine life from plastic pollution. About 10% biodegradation of the copolymer was observed in seawater in 28 days, indicating that it might take less than one year to degrade fully in seawater. It was also degradable in enzyme solution [29].

Glutaric acid is a linear organic dicarboxylic acid which is naturally produced in the human body. It can also be synthesized by ring-opening of butyrolactone and potassium cyanide and can be used as plasticizer and precursor for polyesters and polyamides [30]. Actually, most of the aliphatic polyesters made from even carbon diols and even carbon diacids have weak toughness and slow biodegradation rate due to their high crystallinity. In contrast, aliphatic polyesters containing odd diacids and odd diols tend to have lower crystallinity [21]. If glutaric acid is picked up as dicarboxylic acid to synthesize soft blocks, it is expected to have lower *T*_g_ and to improve biodegradability property more than previously reported PMPS and related ABA type triblock copolymers. Poly(2-methyl-1,3-propylene glutarate) (PMPG) has been reported on in the study for the solubility of polyesters in CO_2_ [31], however, the characteristics and biodegradability of PMPG have not been reported in detail.

In this study, we adopted PMPG as a soft segment for PLA-based triblock copolymers, which can be made from common and easily available monomers, glutaric acid (GA), MP, and L-lactide (LA). The molecular structures, thermal properties, and biodegradation behavior of PMPG and the synthesized triblock copolymer were investigated. The aim of this work was to evaluate the influence of PMPG as a soft central block on the various properties of the PLA-based triblock copolymers, along with comparison with the previously synthesized PMPS-contained ABA type triblock copolyesters.

## 2. Materials and Methods

### 2.1. General Considerations

All the experiments of the polymerizations were carried out in a nitrogen stream using Schlenk techniques. ^1^H NMR (500 MHz) and ^13^C NMR (125 MHz) measurements were performed on a Varian system 500 spectrometer at room temperature. The signals for the residual chloroform (*δ* = 7.26 ppm) and for chloroform-*d* (*δ* = 77.16 ppm) were used for the calibration of the chemical shifts in ^1^H and ^13^C NMR spectra in chloroform-d, respectively. Molecular weight distributions of the obtained polymers were evaluated by gel permeation chromatography (GPC) on a Tosoh GPC system (HLC-8320) equipped with a RI detector at 40 °C using tetrahydrofuran (THF) as an eluent at a flow rate of 1.0 mL/min. GPC traces were calibrated by polystyrene standards. The thermal properties such as melting point (*T*_m_), melting enthalpy (Δ*H*_m_), and glass transition temperature (*T*_g_) of the products were evaluated by differential scanning calorimetry (DSC) on a Seiko DSC 6220 instrument, where the DSC data of the polymers were collected in the second heating scan at a heating rate of 10 °C/min after elimination of thermal history by first heating the sample to 200 °C and cooling to −100 °C at 10 °C/min. The decomposition temperature losing 5% of sample weight (*T*_d5_) was measured by thermogravimetry (TG) on SII Seiko EXSTAR 6000 TG/DTA 6300 in the temperature range of 25–500 °C at a heating rate of 10 °C/min under a nitrogen atmosphere. The tensile tests of the obtained copolymer films were performed by using a Shimadzu EZ-LX HS tensile testing machine at an elongation rate of 5 mm/min. Dumbbell-shaped specimens (width, 4 mm; gauge length, 10 mm; thickness, approximately 0.1 mm) were cut from the copolymer sample sheets for the tensile tests. Each tensile test was repeated twice and averaged.

### 2.2. Materials

2-Methyl-1,3-propanediol (MP) (Tokyo Chemical Industry (TCI), Tokyo, Japan) and glutaric acid (GA) (TCI) were used as received. L-Lactide (LA) (TCI) was purified by sublimation in nitrogen prior to use. THF and toluene (Kanto Chemical, Tokyo, Japan) were distilled from sodium benzophenone in nitrogen before use and were stored over sodium. Tin(II) 2-ethylhexanoate (Sn(Oct)_2_) (Sigma Aldrich, St. Louis, MO, USA) was dried with molecular sieves (3Å) before using.

### 2.3. Synthesis of PMPG

**Scheme 1:**
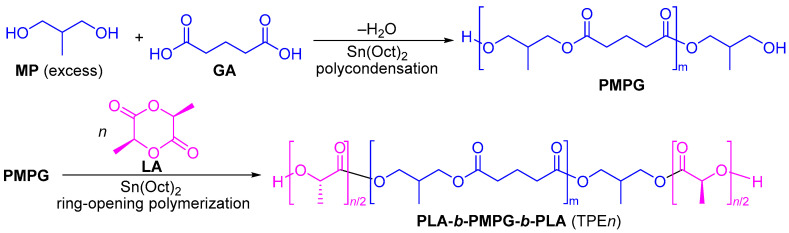
Synthetic scheme of poly(2-methyl-1,3-propylene glutarate) (PMPG) and poly(L-lactide)-*b*-poly(2-methyl-1,3-propylene glutarate)-*b*-poly(L-lactide) (PLA-*b*-PMPG-*b*-PLA) triblock copolymer.

A typical procedure (PMPG-1): PMPG was prepared from the polycondensation of MP (small excess) and GA in the presence of Sn(Oct)_2_ as the catalyst (Scheme 1). At first, a mixture of MP (20.3 g, 0.225 mol), GA (27.06 g, 0.205 mol), and catalyst Sn(Oct)_2_ (0.04 g, 0.1 mmol) was reacted at 180 °C under N_2_ gas atmosphere (1 atm), removing H_2_O using a trap device for 90 min. Then, the mixture was further reacted at 180 °C under reduced pressure below 1.0 mmHg to remove the excess diol for 120 min. The product was dissolved in chloroform and precipitated in excess methanol. The produced polymer was collected and dried in vacuo at room temperature for 2–3 days. The PMPG-1 was obtained in 94% yield as a colorless viscous oil.

### 2.4. Synthesis of PLA-b-PMPG-b-PLA

A typical procedure ([LA]_0_:[PMPG-1]_0_ = 100:1; TPE1, Scheme 1): At first, PMPG-1 (0.16 g, 1.7 × 10^−2^ mmol) was dissolved with 3 mL toluene in a 10 mL Schlenk tube. It took about 1 h of continuous stirring at room temperature to dissolve properly. LA (0.25 g, 1.73 mmol) was added to the solution, and the mixture was heated to 100 °C to dissolve LA completely. Then, Sn(Oct)_2_ (1.6 mg, 0.004 mmol) was added to the mixture to start the LA polymerization. After the reaction at 100 °C for 24 h, acidic methanol was added to the mixture to quench the polymerization, and the resulting copolymer was precipitated in excess methanol. The produced polymer was collected by centrifugation and dried in vacuo at room temperature for 24 h. TPE1 was obtained in 92 wt% yield as a colorless solid.

### 2.5. Formation of Films of the Obtained Polymers

The solution of PLA-*b*-PMPG-*b*-PLA (0.30 g) in THF (4.0 mL) was poured in a Perfluoroalkoxyalkane (PFA) plate. The slow evaporation of the solvent at room temperature under ambient pressure for 3 days followed by further evaporation in vacuo at room temperature for 1 day yielded the self-standing films of the samples.

### 2.6. Enzymatic Biodegradation Test of the Polymers

The enzyme solutions (1 mg/0.02 M buffer) of lipase PS and proteinase K were prepared using 0.02 M phosphate buffer (pH = 8.0). The phosphate buffer (1 mL), 1 mL of H_2_O containing 0.5 mL of enzyme solution, and a sample polymer (10 mg), were placed in a vial. The enzymatic degradations were carried out at 45 °C by shaking the vial for 6 h and 24 h separately. The filtrate of the solution was analyzed by total organic carbon concentration (TOC) measurement by using a TOC analyzer (Shimadzu TOC-VCSH) to evaluate the weight loss of the samples. The degradation test was repeated four times for each sample and the results were averaged.

### 2.7. Biodegradation Test of the Polymers in Seawater

The biodegradation of the obtained polymers in seawater was monitored by biological oxygen demand (BOD) measurements. The seawater was collected at the shoreline of Osaka South Port area by bucket and used within two days. The seawater used for TPE100 and TPE50 was taken on 10 October 2020, and that for PMPG was taken on 13 November 2020. In a typical procedure, a 250 mL BOD testing bottle was charged with polymer sample (30 mg) and 200 mL of the supernatant of seawater, which was equipped to the BOD tester (TAITEC, BOD200F). Calcium hydroxide was used to remove carbon dioxide (CO_2_) evolved from metabolism of the sample by microorganisms from the closed BOD system. The BOD testing bottle was stirred at 27 °C for 28 days while monitoring O_2_ consumption. The control O_2_ consumption volume was subtracted from the observed O_2_ consumption volume of the sample for correction. The theoretical volume of O_2_ consumption for each sample was calculated based on the composition of the polymer samples determined by ^1^H NMR analysis, assuming the complete mineralization of the samples to CO_2_. The % biodegradation values of the samples were simply calculated by the following equation:

% Biodegradation = (consumed O_2_ volume/theoretical volume of O_2_ consumption) × 100

Each biodegradation test was repeated twice and averaged.

## 3. Results

### 3.1. Synthesis of PMPG

In this work, dihydroxyl terminated PMPG were synthesized by a two-stage process of the bulk polycondensation from MP and GA. At first, the condensation of GA with a small excess of MP was conducted at [MP]_0_:[GA]_0_ = 1.1:1 under N_2_ (1 atm) at 180 °C to give MP-terminated oligo(2-methyl-1,3-propylene glutarate)s. Then, the polycondensation was further continued under reduced pressure at 180 °C in the presence of Sn(Oct)_2_ catalyst, removing excess MP (bp 195 °C/1 atm) to produce PMPGs with higher molecular weights (Table 1). The PMPGs were synthesized under different conditions of the reaction time on the 2nd stage for 120, 100, and 50 min, which are named PMPG-1, PMPG-2, and PMPG-3, respectively. All the PMPGs were obtained as colorless viscous oily materials in ≥90% yields.

**Figure 1:**
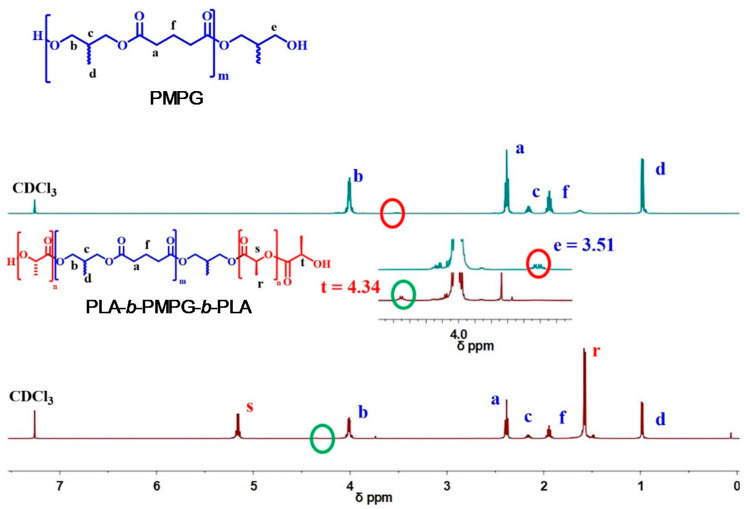
^1^H-NMR spectra of PMPG-1 (**upper**) and PLA-*b*-PMPG-*b*-PLA (TPE100, **lower**).

The 500 MHz ^1^H-NMR spectrum of PMPG-1 is shown in Figure 1 (upper). The signals at *δ* = 0.99 (d), 1.94 (f), 2.16 (c), 2.38 (a), and 4.00 (b) ppm can be assigned to the protons of the repeating units in PMPG [22,29]. In addition, the resonance peak at 3.52 ppm (peak e of PMPG) can be assigned to the α-methylene protons linked to the terminal hydroxyl group, indicating the hydroxy-telechelic structure of PMPG similar to PMPS [29]. The 125 MHz ^13^C-NMR spectrum of the PMPG (Figure S1) also exhibited six sharp resonances assignable to the carbons of the repeating units in PMPG [22].

**Figure 2:**
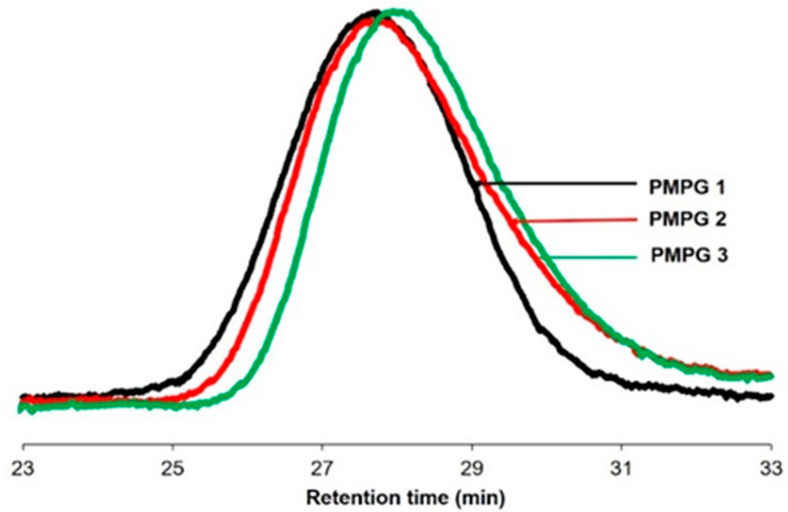
GPC curves of PMPG-1, PMPG-2, and PMPG-3.

Figure 2 represents the GPC curves of the synthesized PMPGs. A monomodal peak in each GPC curve shifted to a smaller retention time with increasing reaction time in the 2nd stage, indicating that increase in the number of the average molecular weight (*M*_n_) of these PMPGs increases the reaction time in the 2nd stage. The polydispersities (*M*_w_/*M*_n_) were 1.56, 1.63, and 1.66 for PMPG-1, 2, and 3, respectively.

**Figure 3:**
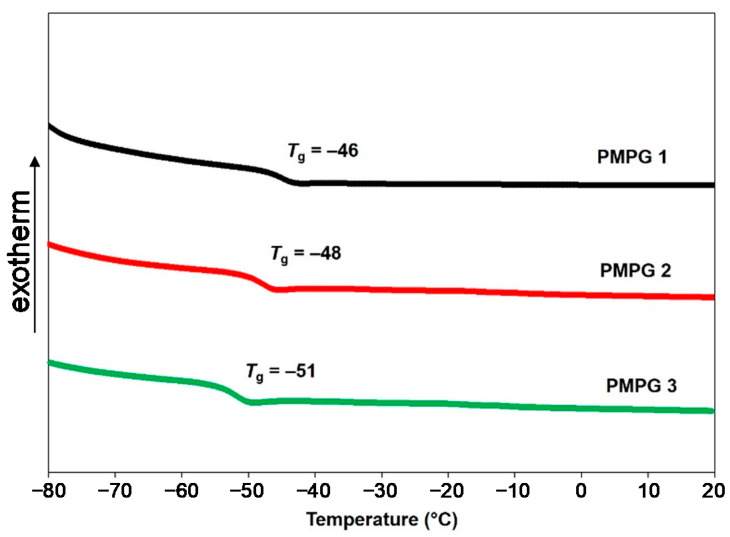
DSC curves of PMPG-1, PMPG-2, and PMPG-3.

The DSC curves of the obtained PMPGs are shown in Figure 3. All the PMPGs exhibited no visible melting peak on their DSC curves. The glass transition of the obtained PMPGs were observed from −46 to −50 °C. Although the *T*_g_ values of the PMPGs tended to increase with the molecular weight, the *T*_g_ of PMPG-1 was as low as −46 °C, resulting in a very sticky transparent polymer at ambient temperature. The thermal decomposition temperatures of the PMPGs were raised by increasing their molecular weights, and the highest *T*_d5_ was observed for PMPG-1 at 377 °C.

### 3.2. Synthesis of PLA-b-PMPG-b-PLA as TPE

The PMPG-1 was applied as a macroinitiator in the LA polymerization at different [LA]_0_:[PMPG-1] feed ratios (Table 2) catalyzed by Sn(Oct)_2_ to synthesize triblock copolymers, PLA-*b*-PMPG-*b*-PLA, as novel biodegradable TPEs. In the sample names, the numbers following “TPE” denote the feed [LA]_0_/[PMPG] molar ratio. The products were characterized by NMR and GPC analysis. 

**Figure 4:**
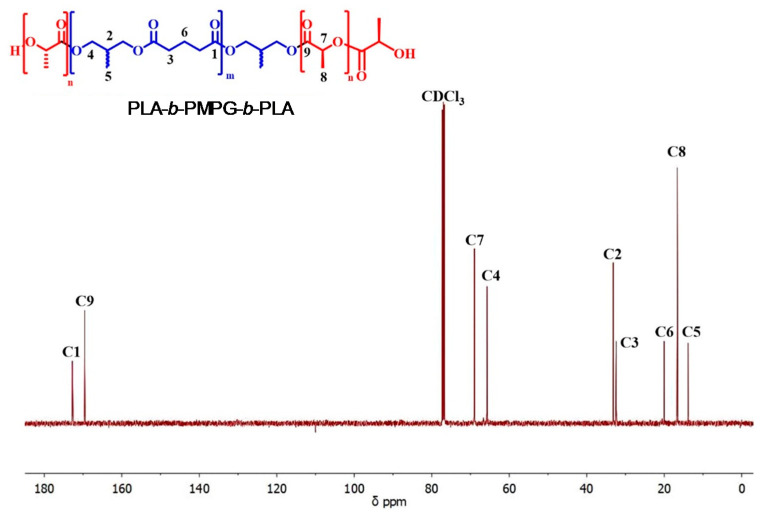
^13^C-NMR spectrum of PLA-*b*-PMPG-*b*-PLA (TPE100).

Figure 1 (lower) shows the ^1^H NMR spectrum of the TPE100. The signal at 3.52 (peak e, Figure 1 (upper)) for the terminal hydroxyl group of the PMPG-1 disappeared, and the new resonance at 4.34 ppm (peak t) for the hydroxyl terminal of PLA appeared in the spectrum of TPE100, indicating that the hydroxyl end of the PMPG-1 initiated the LA polymerization. Apart from the PMPG-peaks, the characteristics peaks of the PLA segment appeared at 5.2 ppm and 1.6 ppm in the spectrum of TPE100, which are the quartet peak (s) of the methine proton in the -OC*H*(CH_3_)CO- unit and the doublet peak of the methyl proton (r), respectively [32]. In Figure 4, the 125 MHz ^13^C-NMR spectrum of TPE100 showed the signals C7 (δ = 69.0 ppm), C8 (δ = 16.6 ppm), and C9 (δ = 169.6 ppm) for the methine, methyl, and carbonyl carbons of the PLA segment, respectively. Apart from the signal for the carbonyl carbon of PMPG, no other signals appeared in the carbonyl region of δ = 169–174 ppm which could be assigned to the mixed comonomer junctions [21]. These results are supported by the observations in the previous reports on the block copolymerization of LA from other aliphatic polyesters [9,13,33,34,35].

**Figure 5:**
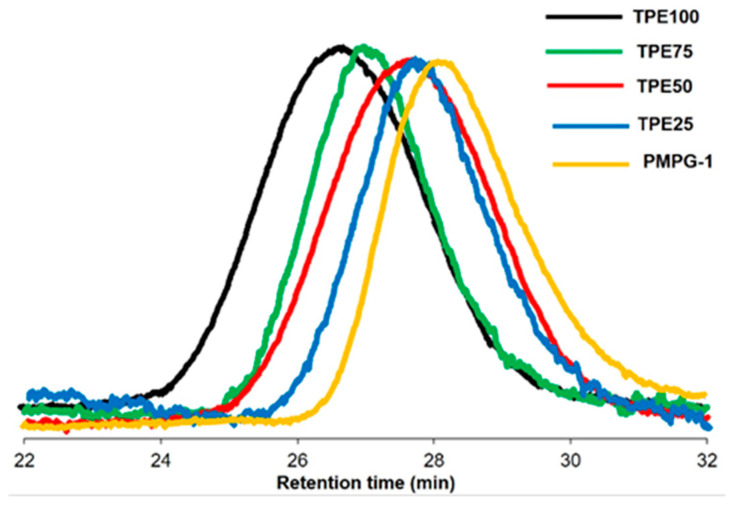
GPC traces of PLA-*b*-PMPG-*b*-PLA samples (TPE100, 75, 50, and 25) and PMPG-1.

Figure 5 represents the GPC traces of PMPG-1 and PLA-*b*-PMPG-*b*-PLAs (TPEs). The peak in the GPC curve shifted from longer retention time to shorter with increase in feed [LA]_0_:[PMPG-1] ratio. The distributions were kept unimodal and relatively narrow in the range of the feed [LA]_0_:[PMPG-1] ratio from 25:1 to 100:1.

Table 2 summarizes the molecular weights and compositions of the obtained TPEs determined by GPC and NMR characterization. The *M*_w_/*M*_n_ of the synthesized copolymers (1.2–1.3) were narrower than that of the macroinitiator (PMPG-1, *M*_w_/*M*_n_ = 1.56) and *M*_n_ of the copolymer increased when the [LA]_0_/[PMPG-1] feed ratio was increased from 25 to 100, suggesting that the molecular weights of the PLA segment can be controlled by the [LA]_0_/[PMPG-1] feed ratio. The molecular weights of the TPEs can also be evaluated from the intensity ratio of the signals for repeating units and the end group in their ^1^H NMR spectra, as in Table 2. The PLA-1 and PLA-2 are the homopoly(L-lactide)s with chain lengths similar to those of the PLA blocks in TPE100 and TPE50, respectively, which were prepared in the previous study for comparison [29].

### 3.3. Thermal Properties of TPEs

**Figure 6:**
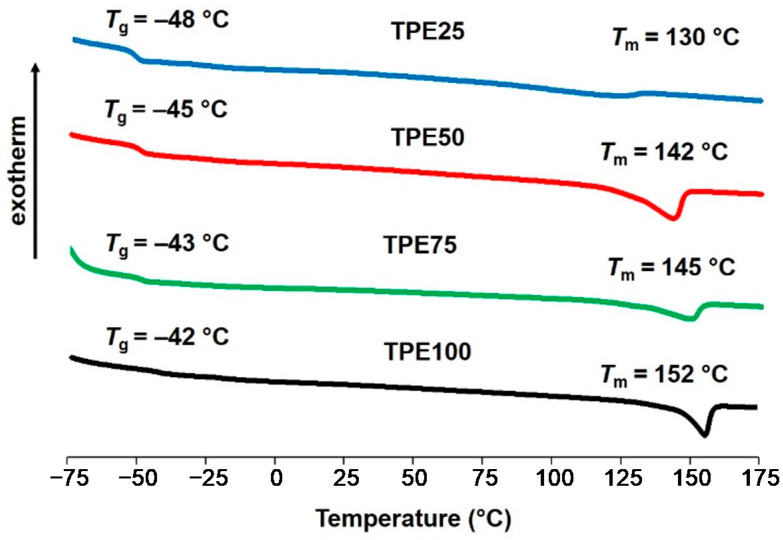
DSC curves of PLA-*b*-PMPG-*b*-PLA samples (TPE100, 75, 50, and 25).

Table 3 summarizes the measured thermal properties of the TPEs. Their DSC curves (2nd heating) are shown in Figure 6. The melting points, *T*_m_, were observed at 152, 145, 142, and 130 °C for the TPE100, 75, 50, and 25, respectively. The *T*_g_ values of the copolymers were observed in a range from −48 to −42 °C. Crystallization peaks were not observed in the heating scan of the TPEs.

**Figure 7:**
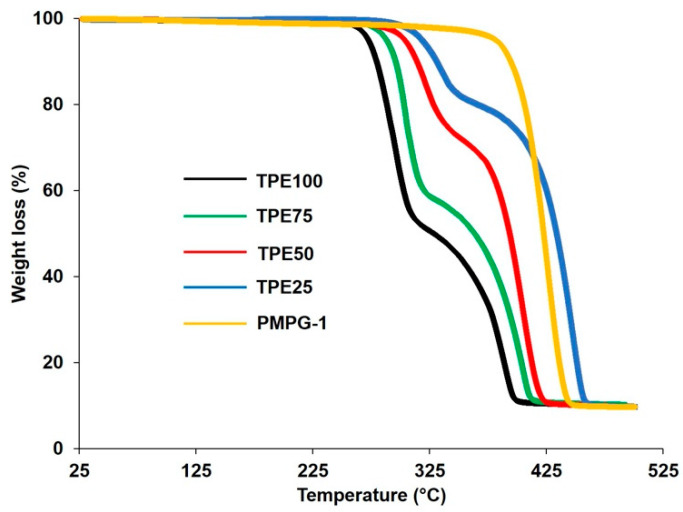
TG curves of PLA-*b*-PMPG-*b*-PLAs (TPE100, 75, 50, and 25) and PMPG-1.

Thermal stability of the PLA-*b*-PMPG-*b*-PLAs copolymers was evaluated by thermogravimetry (TG) analysis (Figure 7). This test illustrated that the thermal degradation process of the triblock copolymers proceeded in two steps. The *T*_d5_ values of the copolymers were 257, 271, 285, and 301 °C for TPE100, 75, 50, and 25, respectively.

### 3.4. Mechanical Properties of the TPEs

**Figure 8:**
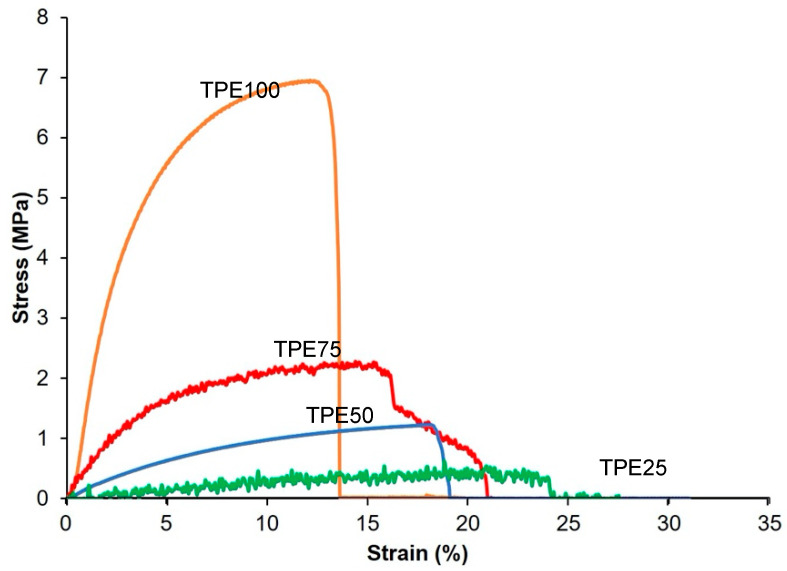
Representative stress–strain curves of the TPE100, TPE75, TPE50, and TPE25.

The mechanical properties of the TPE100, 75, 50, and 25 were evaluated by tensile tests. The self-standing copolymer films (thickness: approximately 0.1 mm) were prepared by solution casting using THF as a solvent and were cut into a nonstandard dumbbell shape. The film of the pure soft segment PMPG could not be formed due to its oily nature. Figure 8 plots the representative stress–strain curves of the copolymers. Table 4 summarizes their tensile properties. The Young’s moduli and tensile strengths of the copolymers tended to increase with the increased LA content of the copolymers. On the other hand, the elongation at break of the TPEs decreased with increased LA content.

### 3.5. Biodegradability of the Synthesized Copolymers

**Figure 9:**
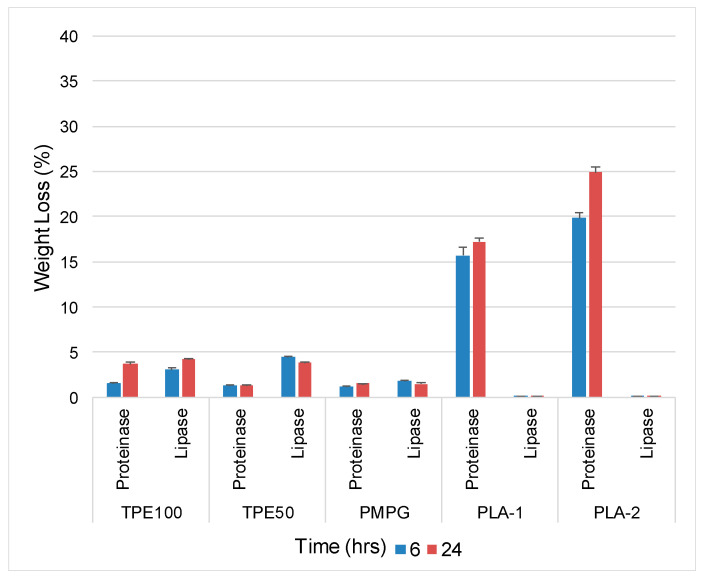
Weight loss % of TPE100, TPE50, PMPG, PLA-1, and PLA-2 in the enzymatic biodegradation using proteinase K and lipase PS.

The biodegradation tests of the polymers were carried out using enzymes proteinase K and lipase PS. Figure 9 shows the weight loss (%) of the TPE100, TPE50, PMPG-1, PLA-1, and PLA-2 (PLA-1: *M*_n_ = 5000 g/mol; PLA-2: *M*_n_ = 3100 g/mol) by the enzymes after 6 and 24 hrs. The results of PLA-1 and PLA-2 are the data from our previous paper for comparison [29]. The PLA-1 and PLA-2 were degraded by 15–25% in a day by proteinase K, while they were hardly degraded in 24 h by Lipase PS [29]. For TPE100, the weight loss increased with time from 6 h to 24 h by both enzymes, while TPE50 and PMPG did not exhibit significant increase of weight loss from 6 h to 24 h. The degradation of PMPG-1 was *ca.* (circa: approximately) 2% within 24 h by both proteinase K and lipase PS. The degradation of TPE100 was *ca.* 4% by both proteinase K and lipase PS, while TPE50 showed degradation of *ca.* 1% and *ca.* 4% by proteinase K and lipase PS, respectively.

**Figure 10:**
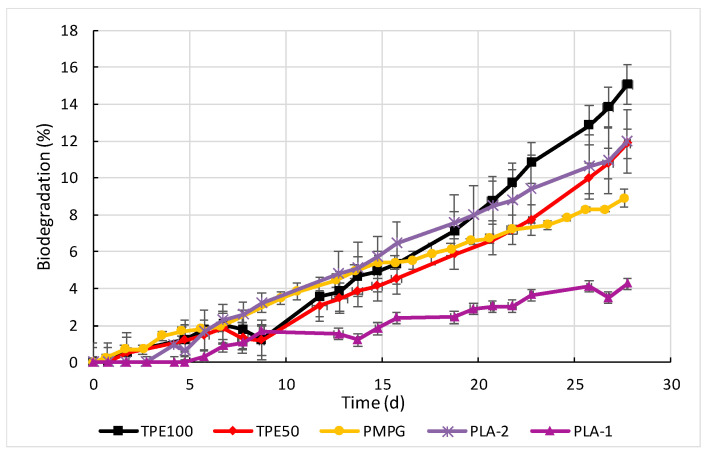
Comparison of seawater biodegradation (%) of the copolymers, PMPG-1, and PLA homopolymers. The data of PLA-1 and PLA-2 are from the previous experiments [29].

The exponential increase of global plastic production has given rise to the issue of marine pollution by plastic debris [36], which has led research works towards the improvement of biodegradability property of the plastic polymeric materials in seawater [37], so we performed the microbial biodegradation of the obtained polymers in seawater. Figure 10 represents the biodegradation (%) of TPE100, TPE50, PMPG-1, PLA-1, and PLA-2 at 27 °C for a total of 28 days in the seawater collected at Osaka port area. This biodegradation of the polymers was monitored by the amount of O_2_ consumed by metabolization (BOD) of the samples by the microorganisms in the seawater. Although the used seawater for PMPG was taken on a different day from that used for TPEs, the results could be roughly compared. The results of PLA-1 and PLA-2 in our previous report [29] were also included in Figure 10 for a rough comparison. For all samples, O_2_ consumption gradually increased with time. The observed biodegradations of TPE100, TPE50, and PMPG after 28 days were around 15%, 12%, and 9%, respectively.

## 4. Discussion

The two-stage polycondensation of MP (small excess) and GA in bulk successfully afforded PMPG as a viscous oily material in high yield. The molecular weights of the obtained PMPG could be modulated by the polymerization time in the second stage. The ^1^H and ^13^C NMR analysis of the obtained PMPGs confirmed their expected structure. DSC analysis of the PMPG did not exhibit melting transition, indicating its amorphous nature. The PMPGs have enough low *T*_g_ at around −50 °C to be used as the soft segment for PLA-conjugated triblock copolymers. The *T*_g_ of the PMPGs were lower than that of PMPS (around −30 °C) [29], reflecting the higher chain mobility of the PMPG than that of the PMPS. In order to obtain higher molecular weight copolymers, PMPG-1 was used as a macroinitiator in the following LA-polymerization to synthesize the following triblock copolymers with enough low *T*_g_ (−46 °C) and relatively high *M*_n_ (9.06 kg mol^−1^). The PMPG-1 showed higher *T*_d5_ at 377 °C than that of the previously reported PMPS (around 360 °C) [29].

The PLA-*b*-PMPG-*b*-PLA triblock copolymers (TPE100, 75, 50, and 25) were synthesized for the first time by the ROP of LA using PMPG-1 as a macroinitiator. The NMR and GPC data of the products strongly supported the successful formation of the expected triblock copolymers. 

**Figure 11:**
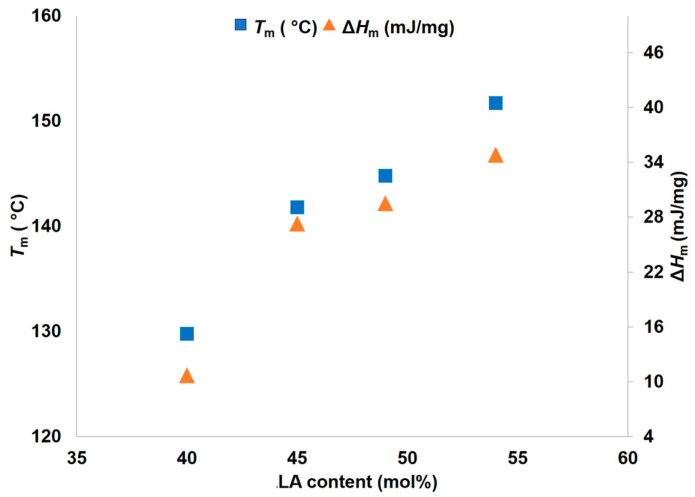
Plots of melting point (*T*_m_; °C) and melting enthalpy (Δ*H*_m_; mJ/mg) vs. LA content of the PLA-*b*-PMPG-*b*-PLA triblock copolymers.

The thermal properties of the obtained triblock copolymers were analyzed by DSC and TG analysis of the samples. DSC analysis of the TPE100, 75, 50, and 25 exhibited their *T*_m_ at 152, 145, 142, and 130 °C, respectively. As PMPG did not show any melting peaks for being amorphous, the melting peaks of the copolymers must come from the melting transition of the PLA crystalline phase. The *T*_m_ and Δ*H*_m_ vs. LA content plots of the TPEs in Figure 11 demonstrates their linear relationship, indicating the controllable thermal properties of the TPEs by LA content. The absence of a crystallization peak in the heating scan of PLA-*b*-PMPG-*b*-PLA suggested their accelerated crystallization in the cooling process.

**Figure 12:**
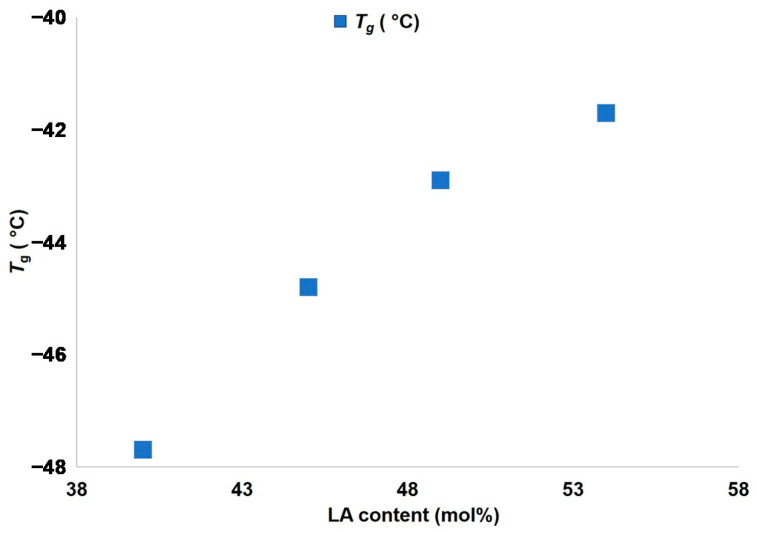
Plots of glass transition temperature (*T*_g_; °C) vs. LA content of the PLA-*b*-PMPG-*b*-PLA triblock copolymers.

The *T*_g_ values of the copolymers below −42 °C correspond to that of the PMPG soft segment. Those of TPE50, 75, and 100 are slightly higher than that of PMPG-1 (−46 °C) because of the presence of a hard PLA segment and are low enough for their use as TPE. Only one *T*_g_ for each TPE indicated the partial miscibility of the PLA and PMPG segments in the amorphous phase of the copolymers. Figure 12 shows the plot of *T*_g_ vs. LA content of the TPEs. The *T*_g_ value also increased with increasing LA content in the copolymers, exhibiting a clear linear relationship. The *T*_g_ values of the PLA-*b*-PMPG-*b*-PLAs are substantially lower than those of PLA-*b*-PMPS-*b*-PLAs (around −20 °C) [29], demonstrating the better cold-resistance of the former. All these data on the thermal properties of the PLA-*b*-PMPG-*b*-PLAs revealed that they have both low *T*_g_ of the soft segment and high *T*_m_ of the hard segment required for thermoplastic elastomers.

The TG analysis of the PLA-*b*-PMPG-*b*-PLAs (Figure 7) showed two step thermal degradation behaviors, where the PLA block degraded at first, then the PMPG block degraded. The copolymers containing shorter PLA blocks showed higher *T*_d5_ values, but lower than that of the PMPG macroinitiator, due to the lower thermal stability of PLA segment than that of PMPG. The residual Sn catalyst could promote the thermal decomposition of PLA [38,39,40].

All the copolymers contain both semicrystalline hard PLA segment and amorphous soft PMPG segment, so they behaved as flexible semicrystalline polymers in their tensile tests, exhibiting elastic deformation. All the copolymers showed significantly higher elongation at break and lower Young modulus than those of the typical PLA [4]. In other words, these TPEs are much softer than PLA because of the incorporated soft segment. The triblock copolymers’ tensile moduli and strengths increased and elongation at break decreased with increasing LA content, most probably due to the increasing physical cross-linking, indicating the controllable physical properties of the TPEs by their compositions. The similar trends are observed in the previously reported PLA-*b*-PMPS-*b*-PLA [29].

The enzymatic degradations of the triblock copolymers and the corresponding homopolymers were performed by two enzymes, proteinase K and Lipase PS, because their specificities to the kinds of polymers are different. The enzymatic degradation rates of polyesters should also greatly depend on their chemical structure, molecular weight, morphology, crystallinity, and so on [41]. Hydrolytic catalyst proteinase K is known for its high activity for PLA degradation [42], and the PLA homopolymers (PLA-1 and -2) were actually degraded up to 25% in 24 h [29]. Lipase PS shows high efficiency towards the hydrolysis of poly(alkanediyl dicarboxylate) [43,44]. The degradation of PMPG by proteinase K (*ca.* 2% after 24 h) was similar to that of PMPS [29]. On the other hand, the degradation of PMPG by lipase PS after 24 h (*ca.* 2%) was considerably lower than PMPS (*ca.* 4%) [29], which could come from the deactivation of lipase PS within 6 h in the PMPG degradation indicated by the similar degradation of PMPG after 6 hrs. The degradation of the copolymers TPE100 and TPE50 by proteinase K (*ca.* 4% and 2% after 24 h for TPE100 and 50, respectively) are lower than homopolymers PLA-1 and -2 considering their compositions, suggesting suppression of degradation by PMPG segment. The degradation of TPE100 and 50 by lipase PS were *ca.* 4% after 24 h, higher than that of PMPG. Although the reason for this observation is not clear yet, we speculate that the deactivation of lipase PS could be suppressed in the TPE100 and 50 systems.

Although there are growing concerns about marine pollution caused by polymer debris, the soft and/or elastic polymer materials that are biodegradable in seawater are still limited compared to hard ones. The biodegradation tests of the triblock copolymers, as well as PMPG, revealed their relatively high biodegradability in seawater. Although the conditions were not the same due to the difference of the seawater used, the biodegradation of PMPG in seawater (about 9% after 28 days) seemed faster than that of PMPS (about 5% after 28 days). The microbial biodegradation of polymers in seawater is affected by the kinds and numbers of microorganisms in it, which may have different substance specificities and activities. Therefore, it is difficult to specify the reason for the higher biodegradability of PMPG than PMPS. However, we speculate that the higher mobility of the polymer chain of PMPG, suggested by its lower *T*_g_ than that of PMPS, could be one of the factors for the faster biodegradation of PMPG. Both TPE100 and TPE50 showed similar biodegradability to that of PMPG. Somewhat higher degradation of TPE100 and TPE50 than that of PMPG could come from the different dates when the seawater was collected. For rough estimation, these materials will be completely degraded within 7–10 months in seawater, assuming linear degradation with time. The seawater biodegradation of the PLA-*b*-PMPG-*b*-PLA and PMPG seems faster than those of typical biodegradable polymers such as poly(butylene succinate) and commercial PLA, and slower than poly(3-hydroxybutyrate) and poly(ε-caprolactone) [45]. Therefore, the PLA-*b*-PMPG-*b*-PLA and PMPG can be regarded as polymers with moderate biodegradability in seawater. The data of PLA-1 and -2 are from our previous study [29], where PLA-2 (about 11% after 28 days) was more degraded than PLA-1 (about 4% after 28 days). Thus, the PLA-*b*-PMPG-*b*-PLAs can be regarded as totally biodegradable polymers in seawater. Some marine bacteria, such as *Comamonas testosteroni Alcaligenes faecalis AE122*, *Marinobacter* sp., *Nocardiopsis aegyptia* sp., and *Shewanella* sp., have been reported to degrade PHB and its derivatives [46,47,48,49,50]. They excrete extracellular enzymes to degrade PHB and metabolize the water-soluble decomposed products as nutrients. The present triblock copolymers and PMPG should most probably be biodegraded in a similar way.

## 5. Conclusions

In this work, triblock copolymers PLA-*b*-PMPG-*b*-PLA with different feed ratios were synthesized by ROP of LA using PMPG as macroinitiator and Sn(Oct)_2_ catalyst for the first time. PMPG was synthesized by polycondensation of easily available monomers, MP and GA. PMPG showed *T*_g_ value at below −46 °C, which was low enough for use as the soft segment of TPE. From DSC analysis, the synthesized triblock copolymers showed *T*_g_ at lower than −40 °C from the PMPG segment and *T*_m_ at 152 to 130 °C from the PLA segment. Finally, from the biodegradation tests, the copolymers and PMPG were found to show relatively high biodegradability in seawater. Thus, the PLA-*b*-PMPG-*b*-PLA can be a potential candidate for practical TPEs with relatively high biodegradability.

## Figures and Tables

**Table 1:** Synthesis of PMPG by the polycondensation of 2-methyl-1,3 propanediol (MP) and glutaric acid (GA) ^a^.

Sample	Time (2nd Stage) (min)	Yield (%)	*M*_n,GPC_^b^ (kg mol^−1^)	*M*_n,NMR_^c^ (kg mol^−1^)	*M*_w_/*M*_n_^b^	*T*_g_^d^ (°C)	*T*_d5_^e^ (°C)
PMPG-1	120	94	9.1	9.7	1.6	−46	377
PMPG-2	100	91	8.3	8.2	1.6	−48	366
PMPG-3	50	90	5.4	5.3	1.7	−51	209

^a^ Reaction conditions, Sn(Oct)_2_ = 0.1 mmol, [MP]_0_:[GA]_0_:[Sn(Oct)_2_] = 1.1:1:0.0005, temperature = 180 °C, time (1st stage) = 1 h, pressure (1st stage) = 1 atm, reduced pressure (2nd stage) = below 1.0 mmHg. ^b^ Determined by gel permeation chromatography (GPC) calibrated with polystyrene standards in tetrahydrofuran (THF). ^c^ Determined by ^1^H-NMR. ^d^ Determined by DSC. ^e^ Decomposition temperature losing 5% of sample weight measured by thermogravimetry (TG).

**Table 2:** The ring-opening polymerization (ROP) of L-lactide (LA) catalyzed by Sn(Oct)_2_ using PMPG-1 as macroinitiator ^a^.

Sample	Feed Ratio ([LA]_0_:[PMPG-1])	Yield (%)	*M*_n,GPC_^b^ (kg mol^−1^)	*M*_n,NMR_^c^ (kg mol^−1^)	*M*_w_/*M*_n_^b^	*F*_LA_^c^ (mol%)
TPE100	100:1	92	20.2	19.7	1.2	54
TPE75	75:1	92	18.7	17.3	1.2	49
TPE50	50:1	92	16.2	15.2	1.3	45
TPE25	25:1	88	14.9	14.0	1.3	40
PLA-1 [29]	50:1 ^d^	97	5.0	5.7	1.4	100
PLA-2 [29]	25:1 ^d^	97	3.1	3.1	1.4	100

^a^ PMPG-1 (*M*_n_ = 9.1 kg mol^−1^, *M*_w_/*M*_n_ = 1.61), Cat = Sn(Oct)_2_ (4 × 10^−3^ mmol), [LA]_0_:[Cat] = 400:1, temperature = 100 °C, time = 24 h. ^b^ Determined by GPC. ^c^ Determined by ^1^H-NMR. ^d^ BnOH was used as an initiator instead of PMPG-1.

**Table 3:** Thermal properties of the synthesized triblock copolymers, PLA-*b*-PMPG-*b*-PLAs.

Sample	Feed Ratio ([LA]_0_:[PMPG-1])	*F*_LA_ (mol%)	*T*_g_^a^ (°C)	*T*_m_^a^ (°C)	*T*_d5_^b^ (°C)
TPE100	100:1	54	−42	152	257
TPE75	75:1	49	−43	145	271
TPE50	50:1	45	−45	142	285
TPE25	25:1	40	−48	130	301

^a^ Determined by DSC at 2nd heating scan. ^b^ Decomposition temperature losing 5% of sample weight determined by TG analysis.

**Table 4:** Mechanical properties of the synthesized triblock copolymers ^a^.

Sample	*F*_LA_ (mol%)	Young Modulus (MPa)	Strength ^b^ (MPa)	Elongation at Break (%)
TPE100	54	207 ± 2	6.2 ± 1.2	14 ± 2
TPE75	49	35.51 ± 0.05	2.0 ± 0.3	18 ± 2
TPE50	45	19.80 ± 0.05	1.3 ± 0.3	21 ± 1
TPE25	40	7 ^c^	0.4 ^c^	23 ^c^

^a^ Determined by tensile tester with elongation rate of 5 mm/min, measured twice. ^b^ Strength is defined as the stress at break. ^c^ Measured once.

## Data Availability

The data presented in this study are available on request from the corresponding author.

## Supplementary Materials

The following are available online at https://www.mdpi.com/2075-1729/11/1/43/s1, Figure S1: ^13^C-NMR spectrum of PMPG-1.
