# Supplementary material for: Synthesis, Properties, and Biodegradability of Thermoplastic Elastomers Made from 2-Methyl-1,3-propanediol, Glutaric Acid and Lactide
Source: Life (Basel). 2021 Jan 12;11(1):43. doi: 10.3390/life11010043 (PMC7828133; doi:10.3390/life11010043)
Supplement: Supplementary file 1 [file life-11-00043-s001.pdf]

life

MDPI

Supplementary Materials

Synthesis, Properties, and Biodegradability of Thermoplastic Elastomers Made from 2-Methyl-1,3-Propanediol, Glutaric Acid and Lactide

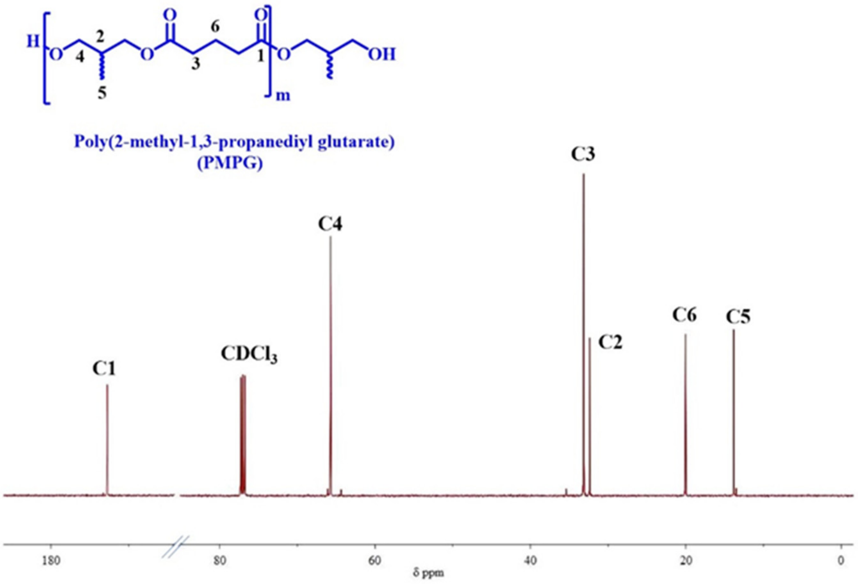

Figure S1. ^{13}C-NMR spectrum of PMPG-1.

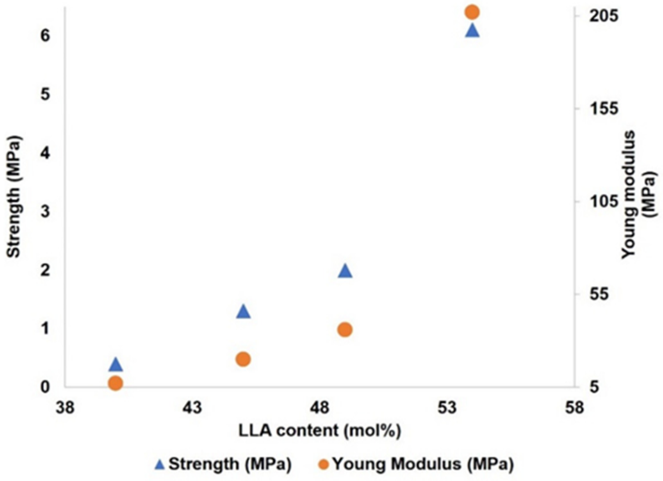

Figure S2. Plots of tensile strength and Young Modulus of the TPEs vs. LA content.

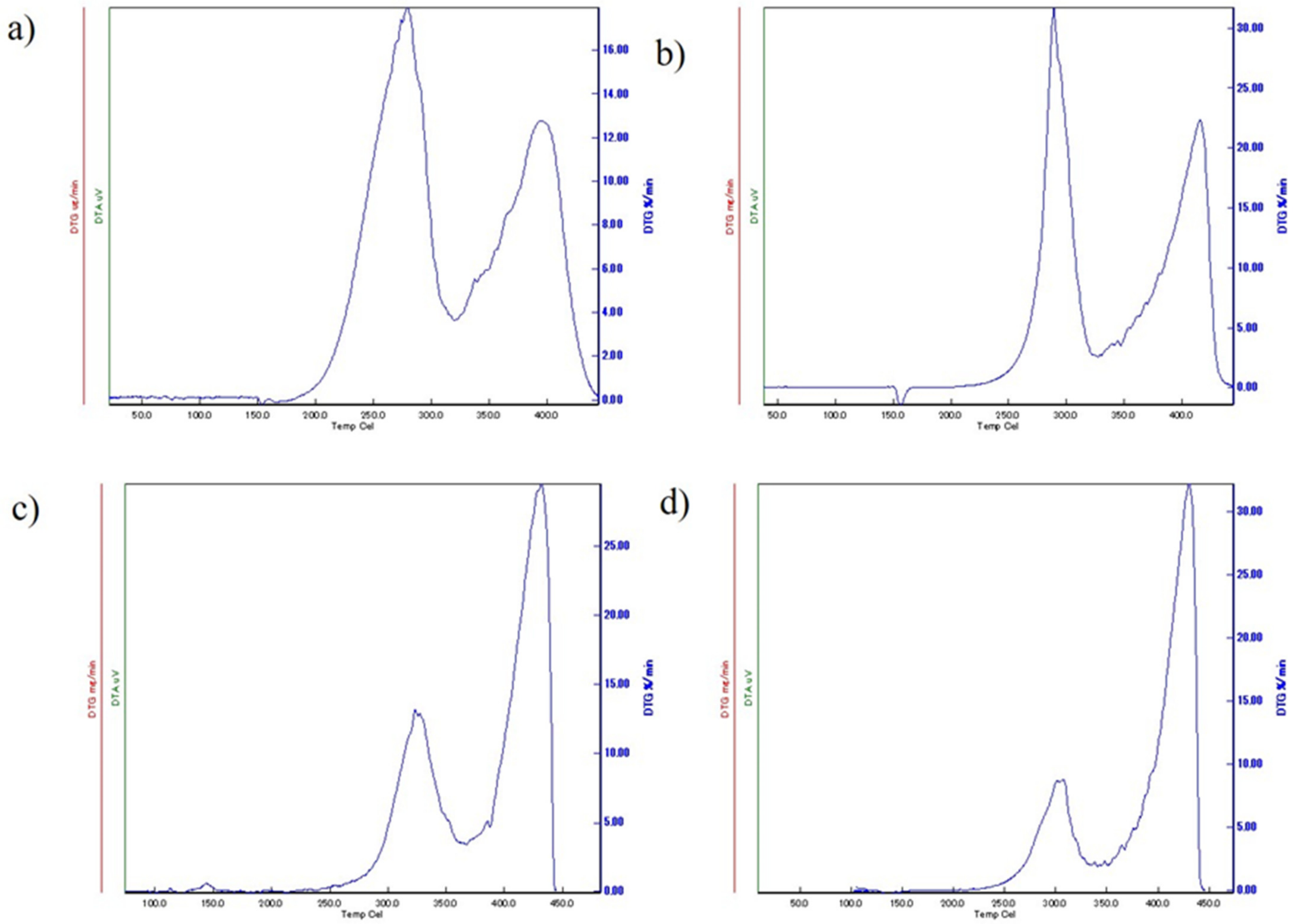

Figure S3. DTG (%/min) curves of (**a**) TPE100, (**b**) TPE75 (**c**) TPE50 (**d**) TPE25.
